# Integration of a fasting-mimicking diet programme in primary care for type 2 diabetes reduces the need for medication and improves glycaemic control: a 12-month randomised controlled trial

**DOI:** 10.1007/s00125-024-06137-0

**Published:** 2024-03-28

**Authors:** Elske L. van den Burg, Marjolein P. Schoonakker, Petra G. van Peet, Elske M. van den Akker-van Marle, Hildo J. Lamb, Valter D. Longo, Mattijs E. Numans, Hanno Pijl

**Affiliations:** 1 Department of Public Health and Primary Care, Leiden University Medical Centre (LUMC), Leiden, the Netherlands; 2 Department of Biomedical Data Sciences, Medical Decision Making, Leiden University Medical Centre (LUMC), Leiden, the Netherlands; 3 Department of Radiology, Leiden University Medical Centre (LUMC), Leiden, the Netherlands; 4 Longevity Institute, Davis School of Gerontology, University of Southern California, Los Angeles, CA USA; 5 FIRC Institute of Molecular Oncology, Milan, Italy; 6 Department of Internal Medicine, Leiden University Medical Centre (LUMC), Leiden, the Netherlands

**Keywords:** Diet, Fasting-mimicking diet, Glucose-lowering medication, HbA_1c_, Lifestyle, Primary care, Randomised controlled trial, Therapy, Type 2 diabetes

## Abstract

**Aims/hypothesis:**

The aim of this study was to evaluate the impact on metabolic control of periodic use of a 5-day fasting-mimicking diet (FMD) programme as an adjunct to usual care in people with type 2 diabetes under regular primary care surveillance.

**Methods:**

In this randomised, controlled, assessor-blinded trial, people with type 2 diabetes using metformin as the only glucose-lowering drug and/or diet for glycaemic control were randomised to receive 5-day cycles of an FMD monthly as an adjunct to regular care by their general practitioner or to receive regular care only. The primary outcomes were changes in glucose-lowering medication (as reflected by the medication effect score) and HbA_1c_ levels after 12 months. Moreover, changes in use of glucose-lowering medication and/or HbA_1c_ levels in individual participants were combined to yield a clinically relevant outcome measure (‘glycaemic management’), which was categorised as improved, stable or deteriorated after 1 year of follow-up. Several secondary outcome measures were also examined, including changes in body weight.

**Results:**

One hundred individuals with type 2 diabetes, age 18–75 years, BMI ≥27 kg/m^2^, were randomised to the FMD group (*n*=51) or the control group (*n*=49). Eight FMD participants and ten control participants were lost to follow-up. Intention-to-treat analyses, using linear mixed models, revealed adjusted estimated treatment effects for the medication effect score (−0.3; 95% CI −0.4, −0.2; *p*<0.001), HbA_1c_ (−3.2 mmol/mol; 95% CI −6.2, −0.2 and −0.3%; 95% CI −0.6, −0.0; *p*=0.04) and body weight (−3.6 kg; 95% CI −5.2, −2.1; *p*<0.001) at 12 months. Glycaemic management improved in 53% of participants using FMD vs 8% of control participants, remained stable in 23% vs 33%, and deteriorated in 23% vs 59% (*p*<0.001).

**Conclusions/interpretation:**

Integration of a monthly FMD programme in regular primary care for people with type 2 diabetes who use metformin as the only glucose-lowering drug and/or diet for glycaemic control reduces the need for glucose-lowering medication, improves HbA_1c_ despite the reduction in medication use, and appears to be safe in routine clinical practice.

**Trial registration:**

ClinicalTrials.gov NCT03811587

**Funding:**

The project was co-funded by Health~Holland, Top Sector Life Sciences & Health, the Dutch Diabetes Foundation and L-Nutra.

**Graphical Abstract:**

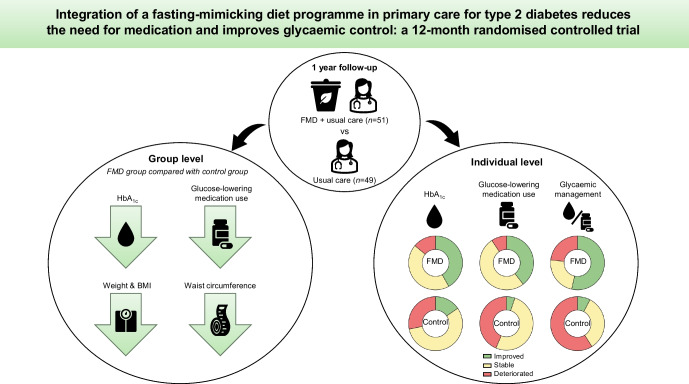

**Supplementary Information:**

The online version of this article (10.1007/s00125-024-06137-0) contains peer-reviewed but unedited supplementary material.


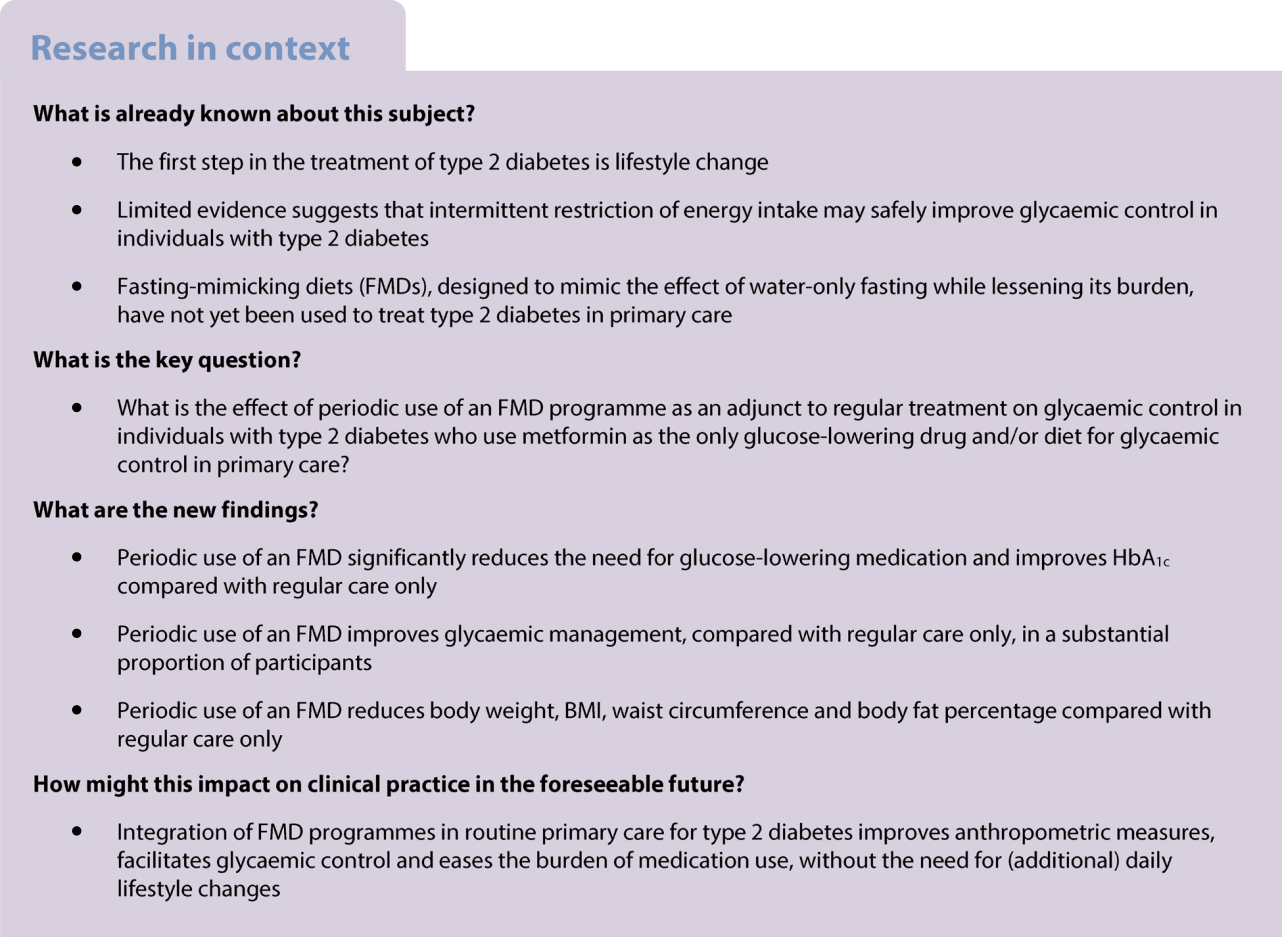


## Introduction

Fasting evokes evolutionarily conserved adaptive hormonal and cellular responses that enhance stress resistance, dampen inflammation and optimise metabolism [1]. Experimental studies have consistently shown robust disease-modifying effects of dietary restriction and intermittent fasting in animal models of chronic disease, including obesity, various cancers, neurodegenerative disorders and diabetes [2–6]. Various methods of intermittent and periodic energy restriction have shown variable effects on glycaemic control in people with type 2 diabetes [7]. Limiting dietary intake to approximately 3560 kJ/day (850 kcal/day) for 12–20 weeks, followed by structural support for weight loss maintenance, facilitates disease remission in people with type 2 diabetes [8–10]. However, severely restricting energy intake for extended periods is burdensome for many people and reduces energy expenditure [11], making weight maintenance a challenge in the long term [12].

Periodic fasting-mimicking diet (FMD) programmes lasting 4–7 consecutive days are designed to mimic the physiological effects of water-only fasting while minimising its burden by allowing individuals to consume light meals during the fasting period and confining it to a limited number of days no more than once a month. These low-energy, plant-based, formula diets are low in sugar and protein, primarily comprising complex carbohydrates and healthy fats [13]. The ‘plant-based’ nature of the diet makes it low in protein, essential amino acids and sugars, and relatively high in fibre and unsaturated fat. Apart from the low energy content, these features are important for the intended fasting-mimicking effects of the diet (i.e. reduction of serum glucose, IGF-1 and insulin, increase in insulin-like growth factor-binding protein-1 and ketone bodies, and reduction in inflammatory markers) [14]. In mice, periodic FMD cycles ameliorate the metabolic anomalies of type 2 diabetes, reverse defects in insulin production [15] and prevent premature death caused by high-fat/high-energy diets [16]. In healthy (non-diabetic) humans, three 5-day cycles of FMD monthly were shown to reduce fat mass, BP, triglyceride levels and fasting glucose, particularly in people with high levels of these risk factors at baseline [17].

The vast majority (90%) of people with type 2 diabetes are under primary care surveillance in the Netherlands [18]. In this study, we evaluated the clinical response to 5-day FMD cycles monthly as an adjunct to regular care in comparison with regular care only in people with type 2 diabetes in a ‘real world’ setting, i.e. under regular primary care surveillance and treatment.

## Methods

### Study design

The Fasting In diabetes Treatment (FIT) trial was designed as a randomised, controlled, assessor-blinded intervention trial conducted at the Leiden University Medical Centre in the Netherlands. The trial was performed according to the principles of the Declaration of Helsinki, in accordance with the Medical Research Involving Human Subjects Act and the standards of Good Clinical Practice. The Medical Research Ethics Committee of Leiden University Medical Centre approved the protocol and amendments. The study was registered as ClinicalTrials.gov NCT03811587, and the study protocol has been published previously [19]. Registration of the trial was initiated prior to the start of the trial; however, online publication occurred after the start of the trial due to a delay within the registration process.

### Participants

In collaboration with general practice centres in the area around Leiden and The Hague, eligible participants under regular primary care surveillance were informed of the study via a letter describing the trial. The participating general practice centres, situated in both the city and the countryside, exhibited diversity, encompassing populations with varying socioeconomic statuses and differing proportions of individuals with a migration background. Race or ethnicity data were not collected, as it was deemed unlikely to influence our results. Individuals with type 2 diabetes, BMI ≥27 kg/m^2^, aged >18 years and <75 years, were eligible. For inclusion, participants had to have an HbA_1c_ > 48 mmol/mol (6.5%) and be treated with lifestyle advice only, or be treated with lifestyle advice plus metformin as the only glucose-lowering drug, irrespective of their HbA_1c_. The exclusion criteria were a recent myocardial infarction (<6 months previously, creatinine clearance <30 ml/min per 1.73m^2^, pregnancy, contraindications for MRI, allergy to ingredients of the diet, history of syncope during caloric restriction or any significant other diseases (at the discretion of the investigator). A total of 129 interested individuals were assessed for eligibility, and 100 were included after providing written informed consent.

### Intervention

Participants were allocated to the FMD group or the control group in computer-generated random sequence via the electronic trial database Castor EDC (https://www.castoredc.com/), which ensured allocation concealment. Permuted block randomisation was performed with block sizes 2 and 4, stratified for sex and weight <100 kg or >100 kg. Sex was determined through self-report during the initial recruitment phase. Due to its nature, blinding of participants to the intervention was impossible, but study research staff who collected outcome data remained unaware of treatment allocation.

Both the control group and the FMD group received usual care through their general practitioner’s office. Usual care entailed 3-monthly clinical and biochemical evaluation, lifestyle advice with the option to consult a dietitian, and adaptation of medication use if necessary. Adaptation of the dose of glucose-lowering medication was completely left to the discretion of the general practitioners, who follow the Dutch guidelines for the treatment of type 2 diabetes in this respect [20]. The study staff did not interfere with usual care in any way. The FMD group received 12 cycles of an FMD on five consecutive days monthly as an adjunct to usual care. Participants were contacted by telephone once during each FMD period to support compliance. The FMD, which is commercially available, comprised complete meal replacement products (see electronic supplementary material [ESM] Table 1). Ingredients were all plant-based and are generally regarded as safe. The energy content and macronutrient composition were as follows: day 1 contained approximately 4600 kJ (approximately 1100 kcal; 10% protein, 56% fat, 34% complex carbohydrate); days 2–5 were identical to each other and provided approximately 3150 kJ (approximately 750 kcal; 9% protein, 44% fat, 47% complex carbohydrate) [19]. The diet of participants who weighed more than 100 kg was supplemented by one choco crisp bar a day (approximately 375 kJ/90 kcal) with similar macronutrient composition. The control group received usual care only. Adherence to the trial regimen was checked verbally every month. We strongly encouraged the participants to complete as many study visits as feasible, even if they decided to quit their assigned treatment, to ensure that missing data were as independent of treatment allocation as possible.

### Outcome measures

Primary and secondary outcomes were measured at baseline, 6 months and 12 months. Specifically, HbA_1c_, total cholesterol, LDL-cholesterol, HDL-cholesterol, triglycerides and high-sensitivity C-reactive protein were measured under fasting conditions. Plasma glucose and insulin concentrations were measured every 30 min over the course of 2 h during an OGTT. Body weight, waist circumference, body fat percentage and BP were also measured. All measurements at 6 and 12 months were performed 3 weeks after the last FMD cycle in those who received FMD.

The primary outcomes were changes in HbA_1c_ and dose of glucose-lowering medication from baseline. The medication effect score (MES) was used as an indirect measure of glucose-lowering drug treatment. The MES of a particular drug dose reflects the decrease in HbA_1c_ that is expected when that specific drug dose is used as monotherapy [21]. It is calculated using the following equation: (actual drug dose/maximum drug dose) × drug-specific adjustment factor. The adjustment factor corresponds to the expected decrease in HbA_1c_ when the drug is used as monotherapy at the maximum recommended dose [22]. The sum of MES values (‘total MES’) attributed to individual drugs in a multidrug regimen thus reflects the maximum HbA_1c_ reduction that may be expected when that specific regimen is used [21]. For instance, a MES of 2.5 for a drug regimen translates to a maximal expected decrease in HbA_1c_ of 2.5%. Therefore, actual HbA_1c_ concentration + total MES was used as a measure of glycaemic control corrected for glucose-lowering medication use [23, 24].

As the response of individual participants (in addition to mean group results) provides valuable insight into the clinical effects of an intervention, we also categorised both outcome measures in each individual participant and used a combined binary outcome to estimate the sample size for the trial (see ESM Methods). For the main analysis, HbA_1c_ was categorised as ‘improved’ when HbA_1c_ was ≥5 mmol/mol (0.5%) lower compared with baseline. It was categorised as ‘deteriorated’ when HbA_1c_ was ≥5 mmol/mol (0.5%) higher compared with baseline. Otherwise, it was categorised as ‘stable’ (Table 1). Any lower dose or discontinuation of metformin compared with baseline was categorised as a ‘decrease’ in the use of glucose-lowering medication. Any higher dose or use of additional glucose-lowering drug was categorised as an ‘increase’ in the use of glucose-lowering medication. When the dose remained the same, drug use was categorised as ‘stable’ (Table 1). As plasma HbA_1c_ concentration and the dose of glucose-lowering drugs mutually influence each other, we combined these parameters reflecting glucose control in individual participants to yield a categorical outcome measure, for which we coined the term ‘glycaemic management’ (Table 1).

**Table 1:** Categories of individual changes at the end of the study compared with baseline for HbA_1c_, glucose-lowering medication use and glycaemic management

Category	Description
HbA_1c_ levels	
Improved	≥5 mmol/mol (0.5%) lower
Stable	<5 mmol/mol (0.5%) higher or lower
Deteriorated	≥5 mmol/mol (0.5%) higher
Use of glucose-lowering medication	
Decreased	Lower dose of metformin or medication stopped
Stable	Stable dose
Increased	Increased dose of metformin and/or use of additional drugs to control glycaemia
Glycaemic management^a^	
Improved	A lower dose or class of glucose-lowering medication with an HbA_1c_ not more than 5 mmol/mol (0.5%) higher at the end of the study compared with baseline; OR: no change in glucose-lowering medication with an HbA_1c_ ≥5 mmol/mol (0.5%) lower at the end of the study compared with baseline
Stable	No change in glucose-lowering medication use and a difference in HbA_1c_ of <5 mmol/mol (0.5%) at the end of the study compared with baseline
Deteriorated	A higher dose or class of glucose-lowering medication at the end of the study compared with baseline; OR: an HbA_1c_ that is ≥5 mmol/mol (0.5%) higher at the end of the study compared with baseline with no change in glucose-lowering medication

^a^The term ‘glycaemic management’ is used to describe the change in HbA_1c_ levels and use of glucose-lowering medication combined

Secondary outcomes were body weight, BMI, total body fat, waist circumference, BP, fasting plasma glucose, insulin and lipid profiles. Furthermore, plasma glucose and insulin concentrations in response to an OGTT were used to calculate the Matsuda index (reflecting insulin sensitivity) and the disposition index (reflecting endogenous insulin secretion) [25–27]. Adverse events were registered according to the Common Terminology Criteria for Adverse Events version 5.0 [28] during two face-to-face visits at 6 and 12 months, or, in the case of serious adverse events, were reported immediately.

### Statistical analysis

Primary and secondary outcomes were summarised using the mean and SD for normally distributed data or median and IQR in case of an asymmetrical distribution. The categorical outcome measures were analysed using *χ*^2^ tests. When the assumptions of the *χ*^2^ test were violated, Fisher’s exact test was used. The treatment effects over time for the primary and secondary continuous outcomes were estimated using linear mixed models for all available data at baseline, 6 months and 12 months. The linear mixed models included fixed effects for time and time-by-arm interaction terms with random effects for individual participants. The models were adjusted for the baseline value of the outcome and for randomisation stratifiers (sex and weight >100 kg) [29]. The Benjamini–Hochberg procedure was used to correct the statistics of the multiple tests of secondary outcomes. An intention-to-treat (ITT) analysis was conducted as well as a per protocol (PP) analysis, including only participants in the FMD group who were compliant with the 12 cycles of FMD. Imputation was not performed, as this could only be applied to the outcome measure, where no power or efficiency would be gained. The last measurement carried forward method was not applied because of the bias it would introduce [30].

As a post hoc analysis, we adjusted the linear mixed models of the primary outcomes by adding a fixed effect for body-weight over time. Moreover, we compared several clinical baseline characteristics of responders and non-responders (with respect to glycaemic management) using independent Student’s *t* tests.

Statistical analyses were performed using Rstudio version 4.3.1 for Windows (http://www.rstudio.com/). Figures were created in GraphPad Prism version 9.0.1 for Windows (https://www.graphpad.com).

## Results

### Trial participants

**Fig. 1:**
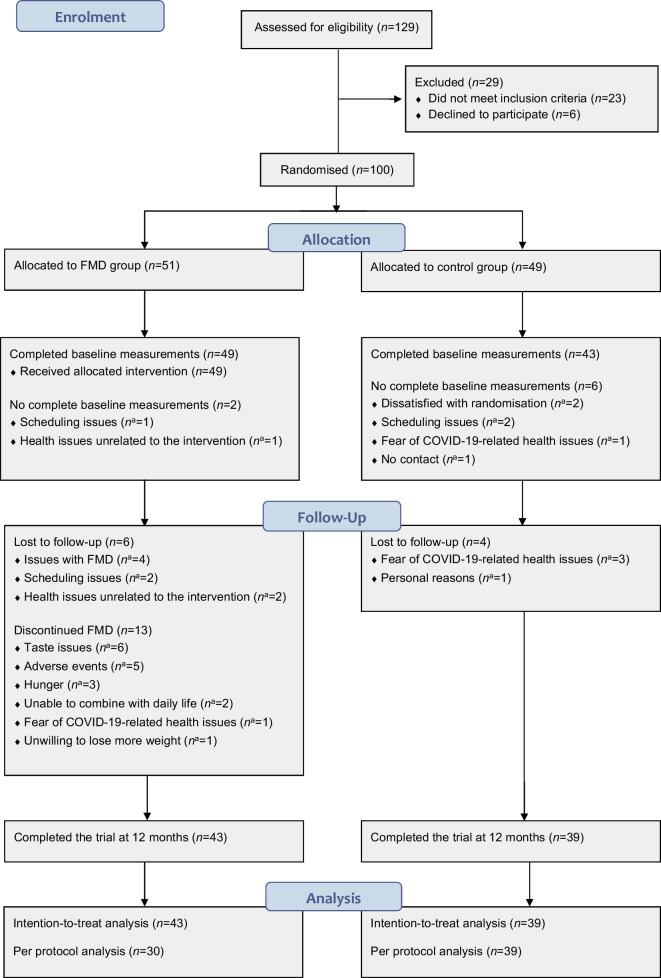
Study flow chart. *n*^a^ indicates the number of participants for whom this was the reason for being lost to follow-up or discontinuing FMD; there may be several reasons per participant

Between 20 November 2018 and 1 July 2020, 129 individuals were assessed for eligibility, of whom 29 were excluded; thus 100 participants were randomly assigned to the FMD group (*n*=51) or the control group (*n*=49) (Fig. 1). Follow-up ended on 5 August 2021.

Two participants in the FMD group and six participants in the control group did not complete baseline measurements. Thus, data for 49 participants using FMD and 43 control participants were available for use in the ITT analysis (Fig. 1). Despite strong encouragement, six non-compliant FMD participants and four control participants could not complete follow-up visits. Indeed, loss to follow-up was primarily due to the inability to complete study visits and unrelated to treatment issues. Moreover, participants who were lost to follow-up were equally distributed among study groups. For these reasons, missing data were assumed to be random. At various time points during the protocol, 13 other participants stopped using the FMD, but agreed to complete follow-up visits (Fig. 1). Demographic and clinical characteristics were similar in both groups at baseline (Table 2). On average, glucose metabolism was well controlled, as indicated by on-target HbA_1c_ values.

**Table 2:** Demographic and baseline characteristics (*n*=92)

	FMD group (*n*=49)	Control group (*n*=43)
Demographic characteristics		
Age (years)	62±8	64±8
Sex		
Male	26 (53)	22 (51)
Female	23 (47)	21 (49)
Level of education		
Low	20 (41)	15 (35)
Medium	13 (27)	13 (30)
High	14 (29)	15 (35)
Country of birth		
the Netherlands	45 (92)	39 (91)
Other	3 (6)	4 (9)
Current smoker	4 (8)	4 (9)
Alcohol use	25 (51)	22 (51)
Medical history		
Time since T2D diagnosis (years)	4 (3–12)	6 (3–10)
T2D complications^a^	7 (14)	6 (14)
Hypertension	35 (71)	29 (67)
Hypercholesterolaemia	39 (80)	26 (60)
History of CVD^b^	8 (16)	5 (12)
Use of glucose-lowering medication		
Metformin	46 (94)	36 (84)
Metformin dose	1000 (500–1700)	1000 (500–1000)
Laboratory measurements		
HbA_1c_ (mmol/mol)	52.2±9.3	53.7±12.2
HbA_1c_ (%)	6.9±0.8	7.1±1.1
Fasting glucose (mmol/l)	8.3±1.9	8.8±1.8
Fasting insulin (pmol/l)	156.0±87.7	146.7±72.1
Lipid spectrum		
Total cholesterol (mmol/l)	4.7±1.0	4.8±0.9
LDL-cholesterol (mmol/l)	2.6±0.9	2.7±0.8
HDL-cholesterol (mmol/l)	1.2±0.3	1.3±0.3
Total cholesterol/HDL-cholesterol ratio	4.0±1.1	3.8±1.0
Triglycerides (mmol/l)	1.8±0.8	1.7±0.7
High-sensitivity CRP (mg/l)	1.6 (0.9–3.3)	2.0 (1.1–4.7)
Anthropometric data		
Weight (kg)	100.5±15.3	99.2±14.3
BMI (kg/m^2^)	31.3 (29.2–35.7)	31.9 (29.8–34.3)
Waist circumference (cm)	112.0±11.7	110.9±9.2
Body fat (%)	37.7±8.1	37.6±7.4
Fat-free mass (kg)	62.4±11.2	62.0±11.9
Systolic BP (mmHg)	140.4±17.0	140.4±14.9
Diastolic BP (mmHg)	84.0±7.1	83.8±7.9

Data are presented as mean ± SD, *n* (%) or median (IQR)

Missing data: One participant in each group did not arrive in fasting condition, therefore fasting glucose and fasting insulin measurements are missing. In the FMD group, one fasting insulin measurement was invalid. Measurements for the plasma lipid spectrum (except HDL-cholesterol) are missing for one participant in the control group due to invalid measurement. Two participants in the FMD group refused to share their level of education; one participant in the FMD group refused to share information on their country of birth

^a^T2D complications include polyneuropathy and retinopathy. There were no cases of nephropathy or diabetic foot

^b^History of CVD includes angina pectoris, myocardial infarction and cerebrovascular events

CRP, C-reactive protein; T2D, type 2 diabetes

### Glycaemic endpoints

#### Glucose-lowering medication use

**Fig. 2:**
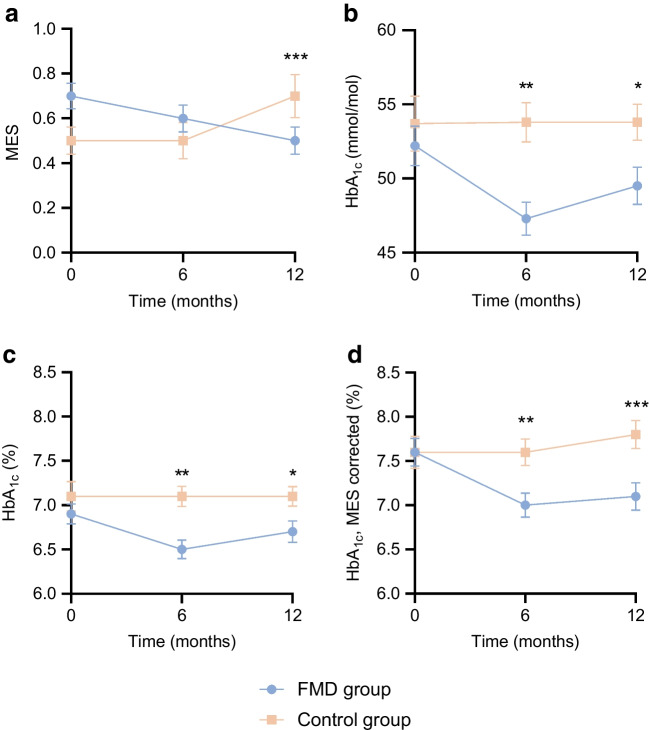
Change over time in total MES, HbA_1c_ and HbA_1c_ corrected for medication use by adding total MES for the FMD group and the control group (ITT analysis). Values are means ± SEM at baseline, 6 months and 12 months. The numbers of participants per timepoint and per group are shown in Table 3. (**a**) Change in MES over time. (**b**) Change in HbA_1c_ (mmol/mol) over time. (**c**) Change in HbA_1c_ (%) over time. (**d**) Change in HbA_1c_ corrected for the total MES over time. *p<0.05; **p<0.01; ***p<0.001 vs the control group

**Fig. 3:**
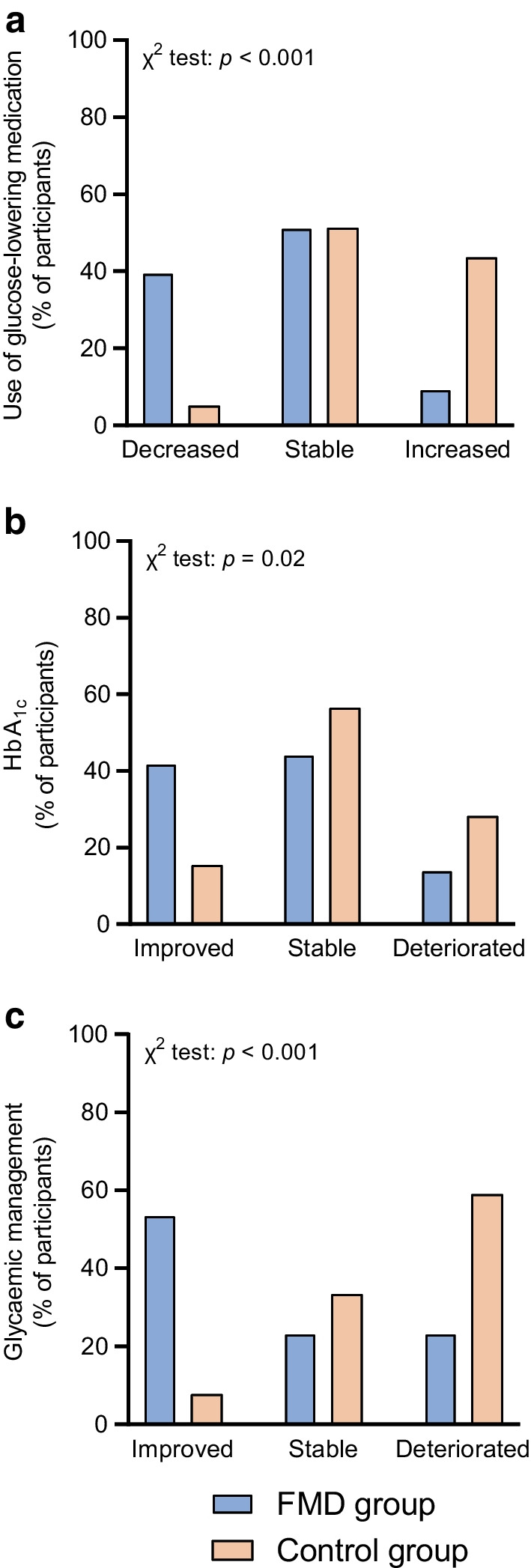
Effect of the FMD on use of glucose-lowering medication and HbA_1c_ levels in participants in the FMD group and the control group at 12 months (ITT analysis). Bars represent the percentage of participants in each category (total *n*=43 in the FMD group vs *n*=39 in the control group). Differences between the FMD group and the control group were evaluated using *χ*^2^ test. The categories are defined in Table 1. (**a**) Change in use of glucose-lowering medication at the end of the study compared with baseline. (**b**) Change in HbA_1c_ at the end of the study compared with baseline. (**c**) Glycaemic management at the end of the study compared with baseline

**Fig. 4:**
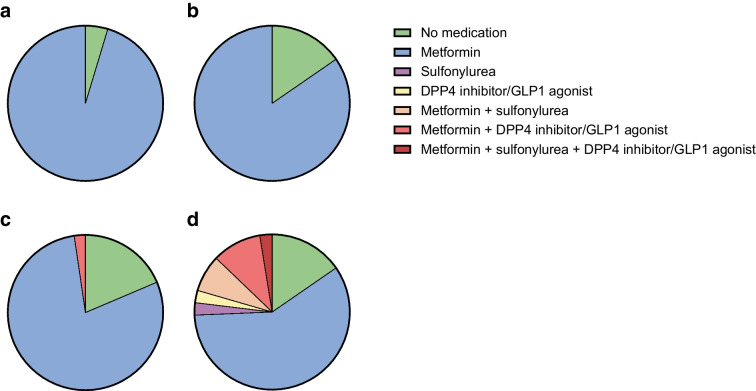
Overview of glucose-lowering medication used at baseline and after 12 months in the FMD group and the control group (ITT analysis). (**a**) Use of glucose-lowering medication in the FMD group at baseline (*n*=43). (**b**) Use of glucose-lowering medication in the control group at baseline (*n*=39). (**c**) Use of glucose-lowering medication in the FMD group after 12 months (*n*=43). (**d**) Use of glucose-lowering medication in the control group after 12 months (*n*=39). DPP4 inhibitor, dipeptidyl peptidase-4 inhibitor; GLP-1 agonist, glucagon-like peptide-1 agonist

The use of glucose-lowering medication, as quantified by the MES, decreased from 0.7 ± 0.4 (mean ± SD) at baseline to 0.5 ± 0.4 at 12 months in the FMD group, but increased from 0.5 ± 0.4 to 0.7 ± 0.6 in the control group, yielding an adjusted estimated treatment effect of −0.3 (95% CI −0.4, −0.2; *p*<0.001) (Table 3 and Fig. 2). The dose of glucose-lowering medication at 12 months was reduced in 40% (*n*=17) of participants in the FMD group and 5% (*n*=2) of control participants, remained stable in 51% (*n*=22) of participants receiving FMD and 51% (*n*=20) of control participants, and increased in 9% (*n*=4) of participants using FMD and 44% (*n*=17) of control participants (*p*<0.001, Fig. 3).The results at 6 months were similar (ESM Fig. 1). Glucose-lowering medication was completely stopped in 16% (*n*=7) of the participants in the FMD group and 5% (*n*=2) of control participants (*p*=0.16), while additional medication was prescribed in 2% (*n*=1) of the FMD group and 26% (*n*=10) of the control group (*p*=0.006, Fig. 4).

**Table 3:** Changes in anthropometric data and plasma metabolic profiles from baseline to 6 months and 12 months in the FMD group and the control group (ITT analysis)

	FMD group		Control group		Adjusted estimated treatment effect (95% CI)	*p* value
	*n*	Mean ± SD	*n*	Mean ± SD		
Primary outcomes						
HbA_1c_ (mmol/mol)						
Baseline	49	52.2±9.3	43	53.7±12.2		
6 months	44	47.3±7.4	37	53.8±8.1	−5.0 (−8.0, −2.0)	<0.01
12 months	43	49.5±8.2	39	53.8±7.6	−3.2 (−6.2, −0.2)	0.04
HbA_1c_ (%)						
Baseline	49	6.9±0.8	43	7.1±1.1		
6 months	44	6.5±0.7	37	7.1±0.7	−0.5 (−0.7, −0.2)	<0.01
12 months	43	6.7±0.8	39	7.1±0.7	−0.3 (−0.6, −0.0)	0.04
MES						
Baseline	49	0.7±0.4	43	0.5±0.4		
6 months	44	0.6±0.4	38	0.5±0.5	−0.1 (−0.2, 0.1)	0.38
12 months	42	0.5±0.4	39	0.7±0.6	−0.3 (−0.4, −0.2)	<0.001
HbA_1c_, MES-corrected (%)						
Baseline	49	7.6±1.1	43	7.6±1.2		
6 months	43	7.0±0.9	36	7.6±0.9	−0.5 (−0.8, −0.2)	<0.01
12 months	42	7.1±1.0	39	7.8±1.0	−0.6 (−0.9, −0.3)	<0.001
Secondary outcomes						
Laboratory measurements						
Fasting glucose (mmol/l)						
Baseline	48	8.3±1.9	42	8.8±1.8		
6 months	42	7.9±1.6	35	9.1±1.9	−0.8 (−1.8, −0.2)	<0.01
12 months	43	8.4±2.1	39	9.0±1.8	−0.4 (−1.0, 0.3)	0.26
Fasting insulin (pmol/l)						
Baseline	47	156.0±87.7	42	146.7±72.1		
6 months	42	155.7±102.2	36	156.2±65.5	−3.4 (−28.9, 22.3)	0.80
12 months	43	164.7±116.9	39	162.6±81.8	−4.7 (−29.7, 20.4)	0.71
Total cholesterol (mmol/l)						
Baseline	49	4.7±1.0	42	4.8±0.9		
6 months	44	4.7±1.0	37	4.9±1.1	−0.0 (−2.3, 0.2)	0.79
12 months	43	4.7±1.0	39	4.8±1.2	0.0 (−0.2, 0.3)	0.87
LDL-cholesterol (mmol/l)						
Baseline	48	2.6±0.9	42	2.7±0.8		
6 months	44	2.6±0.9	37	2.7±1.0	−0.0 (−0.2, 0.2)	0.93
12 months	43	2.6±0.9	39	2.7±1.0	−0.0 (−0.2, 0.2)	0.77
HDL-cholesterol (mmol/l)						
Baseline	49	1.2±0.3	43	1.3±0.3		
6 months	44	1.3±0.3	37	1.3±0.3	0.1 (−0.0, 0.1)	0.09
12 months	43	1.3±0.3	39	1.3±0.3	0.1 (0.0, 0.2)	<0.001
Total cholesterol/HDL-cholesterol ratio						
Baseline	49	4.0±1.1	42	3.8±1.0		
6 months	44	3.8±1.0	37	3.8±0.9	−0.2 (−0.4, 0.1)	0.17
12 months	43	3.7±1.1	39	3.8±1.0	−0.2 (−0.5, 0.0)	0.06
Triglycerides (mmol/l)						
Baseline	49	1.8±0.8	42	1.7±0.7		
6 months	44	1.7±0.7	37	1.8±0.9	−0.2 (−0.4, 0.1)	0.22
12 months	43	1.7±0.7	39	1.8±0.8	−0.1 (−0.4, 0.1)	0.34
High-sensitivity CRP (mg/l)						
Baseline	49	2.6±2.5	43	3.4±3.6		
6 months	44	3.0±4.2	37	2.6±2.1	0.6 (−0.6, 1.9)	0.34
12 months	43	2.5±3.7	39	2.6±2.3	0.1 (−1.1, 1.4)	0.83
Anthropometric data						
Weight (kg)						
Baseline	49	100.5±15.3	43	99.2±14.3		
6 months	44	95.5±14.9	38	98.6±15.0	−3.8 (−5.4, −2.2)	<0.001
12 months	43	95.3±14.5	39	99.4±15.2	−3.6 (−5.2, −2.1)	<0.001
BMI (kg/m^2^)						
Baseline	49	33.0±4.8	43	32.6±3.6		
6 months	44	31.5±4.8	38	32.5±3.7	−1.3 (−1.8, −0.8)	<0.001
12 months	43	31.6±5.0	39	32.6±3.9	−1.2 (−1.7, −0.7)	<0.001
Waist circumference (cm)						
Baseline	49	112.0±11.7	43	110.9±9.2		
6 months	44	108.0±11.3	38	109.8±9.6	−2.6 (−4.5, −0.7)	<0.01
12 months	43	107.7±12.0	39	110.6±9.7	−3.5 (−5.3, −1.6)	<0.001
Body fat (%)						
Baseline	49	37.7±8.1	43	37.6±7.4		
6 months	44	36.3±7.9	38	37.7±7.5	−1.8 (−3.0, −0.7)	<0.01
12 months	43	36.3±8.6	39	37.9±7.5	−2.2 (−3.3, −1.0)	<0.001
Fat-free mass (kg)						
Baseline	49	62.4±11.2	43	62.0±11.9		
6 months	44	60.5±10.1	38	61.4±11.8	−0.7 (−1.6, 0.1)	0.11
12 months	43	60.3±10.0	39	61.8±11.9	−0.3 (−1.1, 0.6)	0.53
Systolic BP (mmHg)						
Baseline	49	140.3±17.0	43	140.4±14.9		
6 months	44	136.5±15.2	38	136.6±16.3	0.6 (−4.9, 6.2)	0.82
12 months	43	137.6±16.7	39	139.0±14.5	−0.4 (−6.0, 5.1)	0.87
Diastolic BP (mmHg)						
Baseline	49	84.0±7.1	43	83.8±7.9		
6 months	44	81.8±7.2	38	82.7±8.4	−0.5 (−3.2, 2.2)	0.72
12 months	43	81.2±5.7	39	82.1±7.0	−0.5 (−3.2, 2.2)	0.74
Insulin sensitivity indices						
Matsuda index						
Baseline	42	1.5±0.8	39	1.5±0.6		
6 months	34	1.7±1.2	23	1.3±0.5	0.4 (0.1, 0.7)	0.03
12 months	39	1.7±1.2	32	1.3±0.5	0.4 (0.1, 0.7)	0.01
Disposition index						
Baseline	42	11.4±6.5	39	11.2±9.5		
6 months	34	11.3±7.3	23	10.4±8.3	0.8 (−2.8, 4.4)	0.68
12 months	39	11.9±10.1	32	10.5±7.3	1.6 (−1.6, 4.8)	0.34

Adjusted estimated treatment effects were calculated using linear mixed models with all available data. The linear mixed models included fixed effects for time and time-by-arm interaction terms with random effects for individual participants. The models were adjusted for the baseline value of the outcome and for randomisation stratifiers (sex and weight >100 kg). *n* = number of participants with data available at each timepoint

CRP, C-reactive protein

#### HbA_1c_

The HbA_1c_ values decreased from 52.2 ± 9.3 mmol/mol (6.9 ± 0.8%) (mean ± SD) at baseline to 49.5 ± 8.2 mmol/mol (6.7 ± 0.8%) at 12 months in the FMD group, and increased from 53.7 ± 12.2 mmol/mol (7.1 ± 1.1%) to 53.8 ± 7.6 mmol/mol (7.1 ± 0.7%) in the control group, yielding adjusted estimated treatment effects of −3.2 mmol/mol (95% CI −8.0, −2.0) and −0.3% (95% CI −0.6, −0.0) (*p*=0.04; Table 3 and Fig. 2). HbA_1c_ was reduced by >5 mmol/mol in 42% (*n*=18) of participants in the FMD group and 15% (*n*=6) of control participants, remained stable in 44% (*n*=19) of participants in the FMD group and 56% (*n*=22) of control participants, and deteriorated in 14% (*n*=6) of FMD participants and 28% (*n*=11) of control participants (*p*=0.02, Fig. 3). The results at 6 months were similar (ESM Fig. 1).

#### Glucose-lowering medication and HbA_1c_ combined

As our primary outcome measures (i.e. use of glucose-lowering medication and HbA_1c_) mutually impact each other, we combined these measures in two distinct ways to better reflect glycaemic control. First, HbA_1c_ (%) corrected for drug treatment by adding the total MES decreased from 7.6 ± 1.1% (mean ± SD) at baseline to 7.1 ± 1.0% at 12 months in the FMD group, but increased from 7.6 ± 1.2% at baseline to 7.8 ± 1.0% in the control group, yielding an adjusted estimated treatment effect of −0.6% (95% CI −0.9, −0.3; *p*<0.001) (Table 3 and Fig. 2). Furthermore, glycaemic management improved in 53% (*n*=23) of participants in the FMD group compared with 8% (*n*=3) of control participants, remained stable in 23% (*n*=10) of participants receiving FMD and 33% (*n*=13) of control participants, and deteriorated in 23% (*n*=10) using FMD and 59% (*n*=23) of control participants (*p*<0.001) (Table 1 and Fig. 3). The results at 6 months were similar (ESM Fig. 1). Two participants in the FMD group could not be formally categorised, as they showed an increase in HbA_1c_ of >5 mmol/mol but they used less glucose-lowering medication after 12 months. We subjectively decided to categorise these participants as deteriorated.

#### Matsuda and disposition index

The Matsuda index and the disposition index were calculated using glucose and insulin data obtained during an OGTT (Table 3 and ESM Fig. 2). The Matsuda index increased from 1.5 ± 0.8 (mean ± SD) at baseline to 1.7 ± 1.2 at 12 months in the FMD group, but decreased from 1.5 ± 0.6 to 1.3 ± 0.5 in the control group, yielding an adjusted estimated treatment effect of +0.4 (95% CI +0.1, +0.7; *p*=0.01; Table 3). The disposition index increased from 11.4 ± 6.5 (mean ± SD) at baseline to 11.9 ± 10.1 at 12 months in the FMD group, but decreased from 11.2 ± 9.5 to 10.5 ± 7.3 in the control group, yielding an adjusted estimated treatment effect of +1.6 (95% CI −1.6, +4.8; *p*=0.34; Table 3).

### Anthropometric data and plasma lipid profiles

There was a significant adjusted estimated treatment effect on body weight (−3.6 kg; 95% CI −5.2, −2.1; *p*<0.001), BMI (−1.2 kg/m^2^; 95% CI −1.7, −0.7; *p*<0.001), waist circumference (−3.5 cm; 95% CI −5.3, −1.6; *p*<0.001) and body fat percentage (−2.2%; 95% CI −3.3, −1.0; *p*<0.001) at 12 months, but no measurable treatment effect on fat-free mass (−0.3 kg; 95% CI −1.1, +0.6; *p*=0.53) (Table 3). Also, there was no treatment effect on systolic BP (−0.4 mmHg; 95% CI −6.0,+5.1; *p*=0.87) or diastolic BP (−0.5 mmHg; 95% CI −3.2, +2.2; *p*=0.74) (Table 3), and antihypertensive drug use was largely unchanged (63% of participants receiving FMD and 79% of control participants used similar antihypertensive medication after 12 months).

There was no treatment effect on plasma lipids, except for an adjusted estimated treatment effect on HDL-cholesterol of +0.1 mmol/l (95% CI 0.0, +0.2, *p*<0.001, Table 3). Use of cholesterol-lowering medication remained stable over 12 months in the vast majority of participants (80% of participants receiving FMD vs 84% of control participants). All variables that were significantly different at 12 months remained so after correction for multiple testing using the Benjamini–Hochberg procedure.

### PP analysis

For the PP analysis, data from FMD participants who were fully compliant with the dietary programme and who finished follow-up (*n*=30) were compared with data from participants in the control group who finished follow-up (*n*=39) (ESM Fig. 3 and ESM Table 2). The mean values for MES and HbA_1c_ changed to a similar extent in the PP and ITT analyses. Medication use decreased in 47% (*n*=14) of participants in the FMD group and 5% (*n*=2) of control participants, remained stable in 47% (*n*=14) of participants in the FMD group and 51% (*n*=20) of control participants, and increased in 7% (*n*=2) of FMD participants and 44% (*n*=17) of control participants (*p*<0.001). HbA_1c_ improved in 50% (*n*=15) of participants in the FMD group and 15% (*n*=6) of control participants, remained stable in 37% (*n*=11) of participants in the FMD group and 56% (*n*=22) of control participants, and deteriorated in 13% (*n*=4) of FMD participants and 28% (*n*=11) of control participants (*p*<0.01). Glycaemic management improved in 63% (*n*=19) of FMD participants compared with 8% (*n*=3) of control participants, remained stable in 17% (*n*=5) of FMD participants compared with 33% (*n*=13) of control participant, and deteriorated in 20% (*n*=6) of FMD participants and 59% (*n*=23) of control participants (*p*<0.001).

### Post hoc analysis

In the post hoc analysis adjusting for body weight over time, the adjusted estimated treatment effect on the MES remained −0.3 (95% CI −0.4, −0.2; *p*<0.001; ESM Table 3). For HbA_1c_, the adjusted estimated treatment effect was −2.0 mmol/mol (95% CI −5.0, +0.9; −0.2%; 95% CI −0.5, +0.1; *p*=0.18). For HbA_1c_ (%) corrected for medication use by adding the total MES, the adjusted estimated treatment effect was −0.4% (95% CI −0.7, −0.1; *p*<0.01).

Moreover, in an attempt to explain the difference between ‘responders’ and ‘non-responders’, we compared several potentially relevant clinical baseline characteristics of both groups, but failed to find significant differences (ESM Table 4). However, baseline HbA_1c_ was non-significantly higher in responders than in non-responders (54.5 ± 10.4 in the FMD group versus 49.0 ± 7.5 in the control group, *p*=0.06).

### Adverse events

The FMD programme caused typical signs of energy deficit (fatigue, headache, dizziness) and nausea in a substantial number of participants during the 5-day intervention, which resolved in the periods between the FMD cycles. Adverse events were registered in 19 FMD and 18 control participants (ESM Table 5). Eight serious adverse events occurred in five FMD participants and no serious adverse events occurred in the control group; none of the serious adverse events were related to the study (ESM Table 6).

## Discussion

We explored the clinical impact of periodic use of an FMD programme as adjunct to usual care for people with type 2 diabetes. The data show that, on average, the group assigned to 12 cycles of five consecutive days of FMD monthly without additional lifestyle advice used significantly less glucose-lowering medication at 12 months, and their HbA_1c_ levels were lower compared with those in the control group. Indeed, the proportion of participants in whom glucose-lowering medication was reduced was eight times higher in the FMD group (40%) than in the control group (5%). Interestingly, HbA_1c_ decreased ≥5 mmol/mol (0.5%) in 42% of participants in the FMD group despite reduced drug use, while this occurred in only 15% of participants in the control group. Moreover, mean body weight, body fat percentage and waist circumference decreased more in participants receiving the FMD programme than in control participants, while fat-free mass did not change. The anthropometric changes were accompanied by an improvement of insulin resistance as reflected by the Matsuda index. Mean changes in BP and plasma lipid profiles did not differ between groups, except for a slightly larger increase in HDL-cholesterol in FMD users.

The potentially confounding effects of medication on HbA_1c_ levels was accounted for by combining the changes in HbA_1c_ and those in glucose-lowering medication. To obtain mean group effects, this was achieved by correcting HbA_1c_ levels for the MES [21]. Moreover, to comprehensively assess the glycaemic control status of individual participants, we constructed a categorical outcome measure that combines HbA_1c_ and the use of glucose-lowering medication, for which we coined the term ‘glycaemic management’. Both measures revealed beneficial effects of the FMD programme on glycaemic control.

The percentage of participants who benefitted from the FMD programme in terms of HbA_1c_ reduction, decrease of glucose-lowering medication or improved glycaemic management was somewhat higher in the PP analysis than in the ITT analysis. However, the differences between analyses were small, and glycaemic management improved even in some participants who discontinued the FMD after just a few cycles. These findings suggest that less frequent dietary intervention may be sufficient to achieve guideline goals. Therefore, further research should aim to define the minimum number and frequency of FMD cycles required for optimal effect.

One of the strengths of this study is that it involved routine monitoring and treatment by general practitioners, which adds to the generalisability of the findings to real-life clinical settings. Indeed, the fact that prescription of glucose-lowering medication was adapted as usual according to Dutch guidelines for the treatment of type 2 diabetes reinforces the notion that the FMD programme will have a similar effect in everyday clinical practice. This approach is likely to yield more realistic and clinically relevant results compared with studies where treatment is tightly controlled according to the study protocol.

A limitation of our study concerns the exclusion of individuals who used glucose-lowering medication other than metformin. We did this because reduction of caloric intake increases the risk of hypoglycaemia in people using sulfonylurea derivatives or insulin (which were the first-choice second- and third-line (drug) treatments, respectively, in the Dutch guidelines at the time the study started). Therefore, prescription of the FMD programme to individuals taking these drugs requires more intense surveillance. In a recent trial examining the same dietary intervention, insulin dose was more than halved and all other glucose-lowering drugs were discontinued during FMD, and participants were required to self-monitor blood glucose concentrations at least four times daily [31]. In this setting, the FMD programme appeared safe, but it was applied to a limited number of participants. Thus, further research is necessary to determine how the FMD programme can be safely applied in individuals who use glucose-lowering medication other than metformin. Furthermore, missing outcome data in the ITT analysis may have caused selection bias, although such data were probably distributed randomly among study groups, as we strongly encouraged people to adhere to (other) protocol instructions even if they discontinued the (dietary) intervention.

The results of three previous studies are in line with our findings. Use of three 5-day cycles of similar composition and timing as used in our trial improved mean anthropometric measures and metabolic control, particularly in obese people with metabolic anomalies at baseline [17], as well as in people with type 2 diabetes [32]. Use of six cycles improved markers of metabolic control in the FMD group but not in a group with similarly timed cycles of a Mediterranean diet in people with type 2 diabetes [31]. The small effect on mean HbA_1c_ levels in our study may be due to the fact that we included participants whose glucose levels were well controlled at baseline. Many studies have shown a strong positive correlation between the mean baseline HbA_1c_ level and its reduction in response to pharmacological intervention [33]. It is quite conceivable that the same is true for lifestyle interventions. The post hoc analysis adjusting for body weight over time showed that there is a direct impact of the FMD on glycaemic outcomes that is independent of weight loss, even though part of its effect is explained by the change in body weight. Earlier animal studies also showed effects of intermittent fasting on glucose and insulin levels that were independent of weight [5, 34].

Our data show that periodic use of an FMD can be a valuable treatment option for people with type 2 diabetes who use metformin as the only glucose-lowering drug and/or diet for glycaemic control. Unfortunately, our post hoc attempt to identify baseline characteristics that predict the response to treatment failed to find significant differences between responders and non-responders, although the HbA_1c_ concentration appeared to be a potential determinant of treatment success, in agreement with data from pharmacological interventions [34]. Post hoc analyses also showed that sex failed to predict treatment response for either treatment success or failure. Based on these results, the FMD programme emerges as a potential treatment option suitable for individuals of both sexes. Future studies should be specifically designed to identify if sex or other determinants can predict success in order to help clinicians decide which patients are most eligible for periodic use of an FMD.

In general, the diet programme was well tolerated, as illustrated by the similar number of (mild to moderately severe) adverse events and dropout rates in the FMD and control groups. However, it is important to note that a variety of (minor) complaints were reported during phone calls at the time participants used the diet, which made five participants discontinue the FMD. It seems prudent to warn people that transient signs of energy deficit (fatigue, dizziness, headache) may occur during FMD periods. Despite these issues, the majority of participants remained motivated and complied with the programme. This indicates that most individuals will eventually be able to sustainably follow an FMD programme in regular care, which is important as the treatment of type 2 diabetes requires lifelong adaptation of dietary habits.

In conclusion, integration of a monthly FMD programme without additional lifestyle advice in regular care for people with type 2 diabetes who use metformin as the only glucose-lowering drug and/or diet for glycaemic control reduces the need for glucose-lowering medication as well as reducing HbA_1c_, and improves anthropometric measures without compromising fat-free mass. Moreover, it appears to be safe in routine clinical practice.

### Supplementary Information

Below is the link to the electronic supplementary material.

Supplementary file1 (PDF 402 KB)

## Data Availability

The datasets generated during and/or analysed in the current study are available upon reasonable request. Requests should be sent to the Fasting In diabetes Treatment (FIT) trial correspondence email (fit@lumc.nl). All proposals requesting data access will need to specify how the data will be used, and all proposals will require the approval of the trial co-investigator team before data release.

## Abbreviations

FMD: Fasting-mimicking diet
ITT: Intention to treat
MES: Medication effect score
PP: Per protocol
