# Supplementary material for: Integration of a fasting-mimicking diet programme in primary care for type 2 diabetes reduces the need for medication and improves glycaemic control: a 12-month randomised controlled trial
Source: Diabetologia. 2024 Mar 28;67(7):1245–59. doi: 10.1007/s00125-024-06137-0 (PMC11153305; doi:10.1007/s00125-024-06137-0)
Supplement: Supplementary file 1 — Supplementary file1 (PDF 402 KB) [file 125_2024_6137_MOESM1_ESM.pdf]

Electronic Supplementary Material

Integration of a fasting-mimicking diet programme in primary care for type 2 diabetes reduces the need for medication and improves glycaemic control: a 12-month randomised controlled trial

Elske L. van den Burg[*1], Marjolein P. Schoonakker[*1], Petra G. van Peet[1], Elske M. van den Akker – van Marle[2], Hildo J. Lamb[3], Valter D. Longo[4,5], Mattijs E. Numans[1], Hanno Pijl[1,6]

Elske L. van den Burg and Marjolein P. Schoonakker are joint first authors.

Contents

Supplementary methods 2

ESM Methods 2

Supplementary tables 4

ESM Table 1. Example meal plan of the fasting-mimicking diet for study participants 4

ESM Table 2. Mean change of anthropometrics and plasma metabolic profiles from baseline to 6 months and 12 months in FMD and control group (per protocol analysis) 5

ESM Table 3. Post-hoc analysis adjusting for weight over time (linear mixed models, intention-to-treat analysis) 7

ESM Table 4. Post-hoc analysis ‘responders’ and ‘non-responders’ (independent Student’s t-test, intention-to-treat analysis) 8

ESM Table 5. Adverse events 9

ESM Table 6. Serious adverse events 9

Supplementary figures 10

ESM Figure 1. Glucose-lowering medication use, HbA_{1c}, and glycaemic management at 6 months (intention-to-treat analysis) 10

ESM Figure 2. Oral glucose tolerance tests of the FMD group and control group at baseline and at 12 months (intention-to-treat analysis) 11

ESM Figure 3. Glucose-lowering medication use, HbA_{1c}, and glycaemic management at 12 months (per protocol analyses) 12

Supplementary methods

ESM Methods

Originally, we defined a binary categorical outcome measure to evaluate the clinical effects of the FMD, using HbA_{1c} levels and glucose-lowering medication use (van den Burg et al, 2020).

The two categories were defined as follows:

- *Successful:* any reduction in glucose-lowering medication use and/or a decrease of HbA_{1c} ≥5 mmol/mol at 12 months compared to baseline.
- *Not successful:* criteria for the category 'successful' are not met.

However, we realised that by classifying treatment outcome as 'successful' or 'not successful' (binary outcome measure), we would fail to recognise the importance of 'stabilising' conditions. Indeed, stable as well as deteriorated metabolic conditions, both reflecting no improvement, classify as treatment failure in the binary outcome model. Thus, we constructed the glycaemic management outcome that is now presented in the manuscript, which appropriately reflects clinically meaningful study results in three categories (improved, stable and deteriorated). We added this outcome via an addendum to the study protocol, which was accepted by the Medical Ethics Research Committee in the LUMC in June 2021, before completion of the follow-up and before access to study data.

The binary outcome measure was originally used to perform the power calculation. Assuming spontaneous improvement of glycaemic control (a 'successful' outcome in the binary outcome measure) in 5% of participants in a group of similar controls(Lean et al, 2018; Adams et al, 2012), inclusion of 45 participants in each group would yield 80% power to detect an absolute difference with FMD of at least 21% (i.e., a 'successful' outcome in 26% of participants using FMD vs 5% of controls, which seems a clinically important difference) at a significance level of 5% using a two-sided binomial test(van den Burg et al, 2020).

Here, we present the data reflecting the impact of the FMD programme on the binary outcome measure:

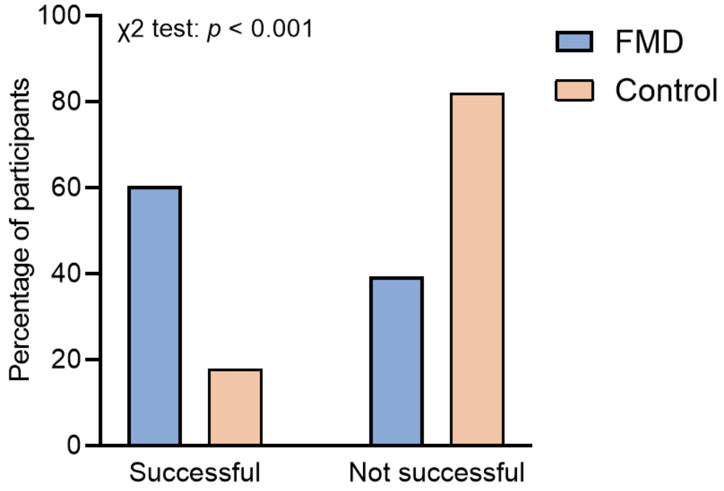

After 12 months, 60% (n=26) of the FMD group was successful compared to 18% (n=7) of controls, while 40% (n=17) of the FMD group was not successful compared to 82% (n=32) of controls (Chi square test, intention-to-treat analysis: p<0.001).

When we drafted the first version of the manuscript, we tried to incorporate both the binary outcome as well as the glycaemic management outcome. However, to improve the readability of the manuscript, we decided not to incorporate the binary outcome in the main manuscript, but to present it in the Electronic Supplementary Material.

References:

- Adams TD, Davidson LE, Litwin SE, et al. (2012) Health benefits of gastric bypass surgery after 6 years. Jama 308(11): 1122-1131. 10.1001/2012.jama.11164
- van den Burg EL, Schoonakker MP, van Peet PG, et al. (2020) Fasting in diabetes treatment (FIT) trial: study protocol for a randomised, controlled, assessor-blinded intervention trial on the effects of intermittent use of a fasting-mimicking diet in patients with type 2 diabetes. BMC Endocr Disord 20(1): 94. 10.1186/s12902-020-00576-7
- Lean MEJ, Leslie WS, Barnes AC, et al. (2018) Primary care-led weight management for remission of type 2 diabetes (DiRECT): an open-label, cluster-randomised trial. Lancet 391(10120): 541-551. 10.1016/S0140-6736(17)33102-1

Supplementary tables

ESM Table 1. Example meal plan of the fasting-mimicking diet for study participants

	Day 1	Day 2	Day 3	Day 4	Day 5
**Breakfast**	Tea<br>Nut bar<br>Algal Oil capsule	Tea<br>Nut bar	Tea<br>Nut bar	Tea<br>Nut bar	Tea<br>Nut bar<br>Algal Oil capsule
**Lunch**	Tomato Soup<br>Olives<br>Kale crackers<br>Vitamin capsule	Tea<br>Mushroom Soup<br>Olives<br>Vitamin capsule	Tea<br>Tomato Soup<br>Kale Crackers<br>Vitamin capsule	Tea<br>Vegetable Soup<br>Olives<br>Vitamin capsule	Tea<br>Tomato Soup<br>Kale Crackers<br>Vitamin capsule
**Afternoon**	Tea<br>Nut bar	Tea<br>Olives	Tea	Tea<br>Olives	Tea
**Dinner**	Minestrone Soup<br>Choco crisp bar<br>Vitamin capsule	Tea<br>Quinoa Mix Soup<br>Choco crisp bar<br>Vitamin capsule	Tea<br>Minestrone Soup<br>Vitamin capsule	Tea<br>Quinoa Mix Soup<br>Choco crisp bar<br>Vitamin capsule	Tea<br>Minestrone Soup<br>Vitamin capsule
**During the day**		Syrup for water flavouring	Syrup for water flavouring	Syrup for water flavouring	Syrup for water flavouring

ESM Table 2. Mean change of anthropometrics and plasma metabolic profiles from baseline to 6 months and 12 months in FMD and control group (per protocol analysis)

	FMD group		Control group		Adjusted estimated treatment effect (95% CI)	p-value
	n	Mean (SD)	*n*	Mean (SD)		
**Primary outcomes**						
HbA_{1c} (mmol/mol)						
Baseline	30	51.0 (8.9)	39	53.9 (12.4)		
6 months	30	45.3 (6.6)	36	53.6 (8.1)	-6.5 (-10.0 to -3.1)	<0.001
12 months	30	47.5 (8.0)	39	53.8 (7.6)	-4.7 (-8.2 to -1.4)	<0.01
HbA_{1c} (%)						
Baseline	30	6.8 (0.8)	39	7.1 (1.1)		
6 months	30	6.3 (0.6)	36	7.1 (0.7)	-0.6 (-0.9 to -0.3)	<0.001
12 months	30	6.5 (0.7)	39	7.1 (0.7)	-0.4 (-0.7 to -0.1)	<0.01
MES						
Baseline	30	0.7 (0.4)	39	0.5 (0.4)		
6 months	29	0.6 (0.4)	36	0.6 (0.5)	-0.1 (-0.2 to 0.1)	0.31
12 months	29	0.4 (0.3)	39	0.7 (0.6)	-0.4 (-0.5 to -0.3)	<0.001
HbA_{1c}, MES corrected (%)						
Baseline	30	7.5 (0.9)	39	7.6 (1.2)		
6 months	29	6.9 (0.8)	35	7.6 (0.9)	-0.6 (-1.0 to -0.3)	<0.001
12 months	29	6.9 (0.9)	39	7.8 (1.0)	-0.7 (-1.1 to -0.4)	<0.001
**Secondary outcomes**						
Laboratory measurements						
Fasting glucose (mmol/l)						
Baseline	29	8.2 (1.7)	38	8.8 (1.9)		
6 months	29	7.7 (1.5)	34	9.0 (1.8)	-1.0 (-1.7 to -0.3)	<0.01
12 months	30	8.2 (2.0)	39	9.0 (1.8)	-0.5 (-1.2 to 0.2)	0.17
Fasting insulin (pmol/l)						
Baseline	28	162.7 (77.7)	38	146.5 (73.3)		
6 months	29	155.3 (110.0)	35	157.6 (66.0)	-7.8 (-37.0 to 21.7)	0.61
12 months	30	165.7 (125.2)	39	162.6 (81.8)	-6.3 (-34.7 to 22.5)	0.67
Cholesterol (mmol/l)						
Baseline	30	4.8 (1.0)	38	4.8 (1.0)		
6 months	30	4.9 (1.1)	36	4.9 (1.1)	0.0 (-0.3 to 0.3)	0.96
12 months	30	4.9 (1.0)	39	4.8 (1.2)	0.0 (-0.2 to 0.3)	0.62
LDL Cholesterol (mmol/l)						
Baseline	29	2.7 (0.9)	38	2.7 (0.8)		
6 months	30	2.8 (0.9)	36	2.7 (1.0)	0.0 (-0.2 to 0.3)	0.74
12 months	30	2.7 (0.9)	39	2.7 (1.0)	0.0 (-0.2 to 0.3)	0.73
HDL Cholesterol (mmol/l)						
Baseline	30	1.2 (0.2)	39	1.3 (0.3)		
6 months	30	1.3 (0.3)	36	1.3 (0.3)	0.1 (0.0 to 0.1)	0.03
12 months	30	1.3 (0.3)	39	1.3 (0.3)	0.1 (0.1 to 0.2)	<0.001
Cholesterol/HDL ratio						
Baseline	30	4.1 (1.2)	38	3.7 (1.0)		
6 months	30	3.9 (1.0)	36	3.8 (0.9)	-0.2 (-0.5 to 0.1)	0.17
12 months	30	3.9 (1.1)	39	3.8 (1.0)	-0.2 (-0.5 to 0.0)	0.10
Triglycerides (mmol/l)						
Baseline	30	1.9 (0.9)	38	1.7 (0.7)		
6 months	30	1.8 (0.8)	36	1.9 (0.9)	-0.2 (-0.5 to 0.1)	0.18
12 months	30	1.8 (0.8)	39	1.8 (0.8)	-0.2 (-0.5 to 0.1)	0.23
High sensitive CRP (mg/l)						
Baseline	30	2.7 (2.8)	39	3.4 (3.6)		

6 months	30	2.8 (3.8)	36	2.7 (2.1)	0.5 (-0.7 to 1.7)	0.44
12 months	30	2.0 (2.1)	39	2.6 (2.3)	-0.4 (-1.6 to 0.8)	0.48
Anthropometrics						
Weight (kg)						
Baseline	30	99.1 (10.5)	39	99.0 (14.8)		
6 months	30	94.1 (10.1)	37	99.0 (15.1)	-4.9 (-6.7 to -3.2)	<0.001
12 months	30	94.6 (10.4)	39	99.4 (15.2)	-4.8 (-6.5 to -3.1)	<0.001
BMI (kg/m^2)						
Baseline	30	32.9 (4.7)	39	32.5 (3.5)		
6 months	30	31.3 (4.6)	37	32.6 (3.7)	-1.7 (-2.2 to -1.1)	<0.001
12 months	30	31.5 (4.8)	39	32.6 (3.9)	-1.6 (-2.2 to -1.0)	<0.001
Waist circumference (cm)						
Baseline	30	111.3 (9.7)	39	110.3 (9.3)		
6 months	30	107.3 (10.2)	37	110.0 (9.6)	-3.5 (-5.5 to -1.6)	<0.001
12 months	30	106.8 (10.3)	39	110.6 (9.7)	-4.5 (-6.4 to -2.5)	<0.001
Body fat (%)						
Baseline	30	37.6 (8.2)	39	37.3 (7.1)		
6 months	30	35.7 (8.5)	37	37.6 (7.6)	-2.1 (-3.3 to -0.9)	<0.001
12 months	30	35.6 (9.0)	39	37.9 (7.5)	-2.6 (-3.8 to -1.4)	<0.001
Fat free mass (kg)						
Baseline	30	61.5 (7.9)	39	62.2 (12.1)		
6 months	30	60.1 (7.5)	37	61.7 (11.8)	-1.1 (-1.8 to -0.3)	<0.01
12 months	30	60.5 (7.8)	39	61.8 (11.9)	-0.5 (-1.3 to 0.2)	0.17
Systolic blood pressure (mmHg)						
Baseline	30	141.3 (19.3)	39	141.1 (15.3)		
6 months	30	137.7 (16.1)	37	137.1 (16.3)	0.8 (-5.5 to 7.1)	0.80
12 months	30	139.4 (18.2)	39	139.0 (14.5)	0.4 (-5.8 to 6.7)	0.89
Diastolic blood pressure (mmHg)						
Baseline	30	84.2 (6.9)	39	84.0 (7.9)		
6 months	30	81.8 (7.5)	37	82.7 (8.5)	-0.9 (-3.9 to 2.2)	0.59
12 months	30	81.5 (6.1)	39	82.1 (7.0)	-0.6 (-3.6 to 2.5)	0.71
Insulin sensitivity indices						
Matsuda Index						
Baseline	25	1.4 (0.7)	36	1.5 (0.6)		
6 months	24	1.9 (1.4)	23	1.3 (0.5)	0.5 (0.2 to 0.9)	<0.01
12 months	28	1.8 (1.3)	32	1.3 (0.5)	0.5 (0.2 to 0.8)	<0.01
Disposition Index						
Baseline	25	10.3 (5.6)	36	11.6 (9.7)		
6 months	24	11.2 (7.7)	23	10.4 (8.3)	0.9 (-3.1 to 5.0)	0.65
12 months	28	12.2 (11.2)	32	10.5 (7.3)	2.4 (-1.3 to 6.1)	0.20

Adjusted estimated effects were calculated with linear mixed models using all available per protocol data. The linear mixed models included fixed effects for time and time-by-arm interaction terms with random effects for individual participants. Models were adjusted for baseline values and randomisation stratifiers.

n = Number of participants with data available at each timepoint.

BMI=body mass index. CI=confidence interval. CRP=C-reactive protein. FMD=fasting-mimicking diet. HbA$_{1c}$=glycated haemoglobin. SD=standard deviation.

ESM Table 3. Post-hoc analysis adjusting for weight over time (linear mixed models, intention-to-treat analysis)

	**FMD group**		**Control group**		**Adjusted estimated treatment effect (95% CI)**	**p-value**
	n	**Mean (SD)**	***n***	**Mean (SD)**		
**Primary outcomes**						
HbA_{1c} (mmol/mol)						
Baseline	49	52.2 (9.3)	43	53.7 (12.2)		
6 months	44	47.3 (7.4)	37	53.8 (8.1)	-3.7 (-6.7 to -0.8)	0.01
12 months	43	49.5 (8.2)	39	53.8 (7.6)	-2.0 (-5.0 to 0.9)	0.18
HbA_{1c} (%)						
Baseline	49	6.9 (0.8)	43	7.1 (1.1)		
6 months	44	6.5 (0.7)	37	7.1 (0.7)	-0.3 (-0.6 to -0.1)	0.01
12 months	43	6.7 (0.8)	39	7.1 (0.7)	-0.2 (-0.5 to 0.1)	0.18
MES						
Baseline	49	0.7 (0.4)	43	0.5 (0.4)		
6 months	44	0.6 (0.4)	38	0.5 (0.5)	-0.0 (-0.2 to 0.1)	0.48
12 months	42	0.5 (0.4)	39	0.7 (0.6)	-0.3 (-0.4 to -0.2)	<0.001
HbA_{1c}, MES corrected (%)						
Baseline	49	7.6 (1.1)	43	7.6 (1.2)		
6 months	43	7.0 (0.9)	36	7.6 (0.9)	-0.3 (-0.6 to -0.1)	0.03
12 months	42	7.1 (1.0)	39	7.8 (1.0)	-0.4 (-0.7 to -0.1)	<0.01

Adjusted estimated treatment effects were calculated using the linear mixed models used for the main analyses in Table 2, with an additional adjustment for bodyweight over time.

CI=confidence interval. CRP=C-reactive protein. FMD=fasting-mimicking diet. HbA_{1c}=glycated haemoglobin. SD=standard deviation.

ESM Table 4. Post-hoc analysis ‘responders’ and ‘non-responders’ (independent Student’s t-test, intention-to-treat analysis)

	**Responders (*n*=23)**	**Non-responders (*n*=20)**	**p-value**
**Demographics**			
Age (years), mean ± SD	65.2 ± 8.1	62.5 ± 7.9	0.28
Sex, *n* (%)			1.00
Male	11 (47.8)	10 (50.0)	
Female	12 (52.2)	10 (50.0)	
**Glycemic control**			
HbA_{1c} (mmol/mol), mean ± SD	54.5 ± 10.4	49.0 ± 7.5	0.06
HbA_{1c} (%), mean ± SD	7.1 ± 1.0	6.6 ± 0.7	0.06
Use of glucose-lowering medication			
Metformin, *n* (%)	22 (95.7)	19 (95.0)	1.00
**Anthropometrics**			
Weight (kg), mean ± SD	99.8 ± 15.0	97.4 ± 12.5	0.57
BMI (kg/m^2), median (IQR)	31.0 (30.1-34.4)	31.1 (28.7-35.8)	0.57
Waist circumference (cm), mean ± SD	112.5 ± 12.5	109.8 ± 9.4	0.43

Responders were participants who improved in glycaemic management, while non-responders were participants who remained stable or who deteriorated in glycaemic management (Fig. 3).

BMI=body mass index. HbA_{1c}=glycated haemoglobin. *n*=number of participants. SD=standard deviation.

ESM Table 5. Adverse events

	Reported at regular appointments (6 months and 12 months)		In FMD period
**Symptom**	**FMD**	**Control**	
Bloating	1		2
Concentration impairment			2
Constipation	1		2
Diarrhoea	1		9
Dizziness	1	1	12
Dyspepsia			2
Fatigue	1	1	15
Flatulence			2
Generalised muscle weakness			3
Headache	1	1	11
Irritability			1
Muscle cramp			1
Nausea			10
Presyncope			4
Vomiting			4
Other adverse events	13	15	57
of which			
Upper respiratory tract symptoms	1	6	18
Musculoskeletal	5	8	11

Number of unique symptoms, there may be several symptoms per participant. At regular appointments (6 months and 12 months), participants were asked for occurrences of adverse events. Between regular appointments, phone calls to participants of the FMD group during the FMD days were made and the mentioned adverse events were reported. Adverse events were reported following the Common Terminology Criteria for Adverse Events (CTCAE) version 5.0.
FMD = fasting-mimicking diet

ESM Table 6. Serious adverse events

Event	Number of occurrences
Hospitalization for anaphylactic shock	1*
Hospitalization for cardioversion	1
Hospitalization for cerebrovascular accident	1
Hospitalization for decongestive heart failure	1
Hospitalization for fever due to viral infection	1
Hospitalization for heart surgery	1
Hospitalization for hip replacement	1
Hospitalization for surgery of carcinoma	1

Number of unique events, there may be several events per participant.
*which did not take place during the 5-days of the fasting-mimicking diet

Supplementary figures

ESM Figure 1. Glucose-lowering medication use, HbA_{1c}, and glycaemic management at 6 months (intention-to-treat analysis)

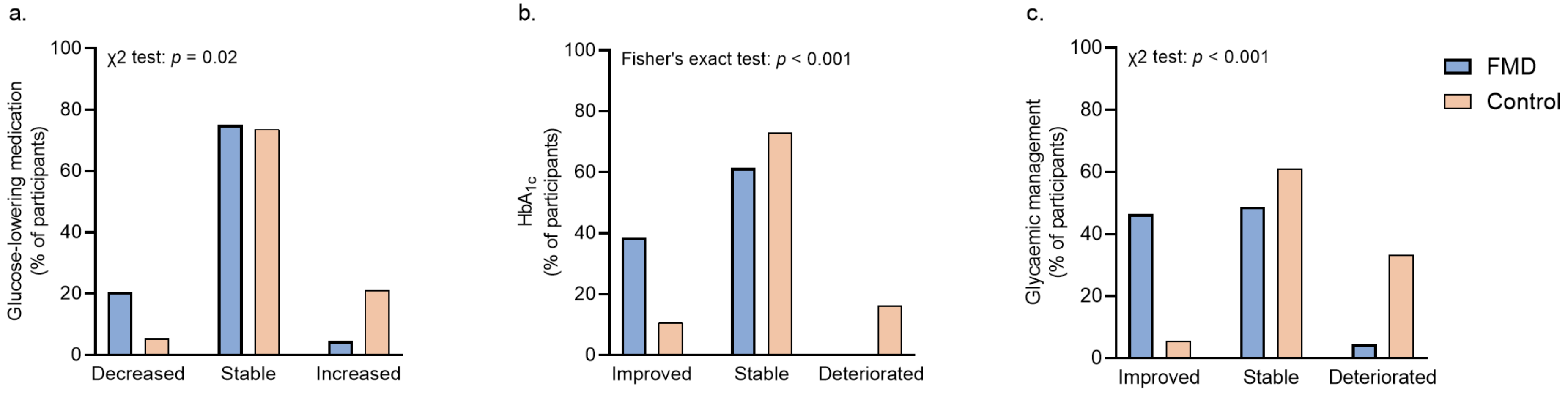

Plotted bars represent percentage of participants. Differences between FMD group and control group were evaluated using the Fisher's exact test or the Chi square test. Number of participants with data available at baseline and 12 months were used for each outcome.

(A) Change in glucose-lowering medication, with decrease defined as a lower dose, stable as no change and increased as higher dose or other class of glucose-lowering medication at the end of the study compared to baseline. FMD *n*=44, controls *n*=38

(B) Change in HbA_{1c}, defined as follows; Improved: an HbA_{1c} that is ≥0.5 % (≥5 mmol/mol) lower at the end of the study compared to baseline. Stable: a change in HbA_{1c} of <0.5 % (<5 mmol/mol) at the end of the study compared to baseline. Deteriorated: an HbA_{1c} that is ≥0.5 % (≥5 mmol/mol) higher at the end of the study compared to baseline. FMD *n*=44, controls *n*=37

(C) Glycaemic management, defined as follows; Improved: a lower dose or class of glucose-lowering medication with an HbA_{1c} not more than 0.5 % (5 mmol/mol) higher at the end of the study compared to baseline or; no change in glucose-lowering medication with an HbA_{1c} that is ≥0.5 % (≥5 mmol/mol) lower at the end of the study compared to baseline. Stable: no change in glucose-lowering medication use and a difference in HbA_{1c} of <0.5 % (<5 mmol/mol) at the end of the study compared to baseline. Deteriorated: a higher dose or class of glucose-lowering medication at the end of the study compared to baseline or; an HbA_{1c} that is ≥0.5 % (≥5 mmol/mol) higher at the end of the study compared to baseline with no change in glucose-lowering medication. FMD *n*=43, controls *n*=36.

FMD=fasting-mimicking diet. HbA_{1c}=glycated haemoglobin.

ESM Figure 2. Oral glucose tolerance tests of the FMD group and control group at baseline and at 12 months (intention-to-treat analysis)

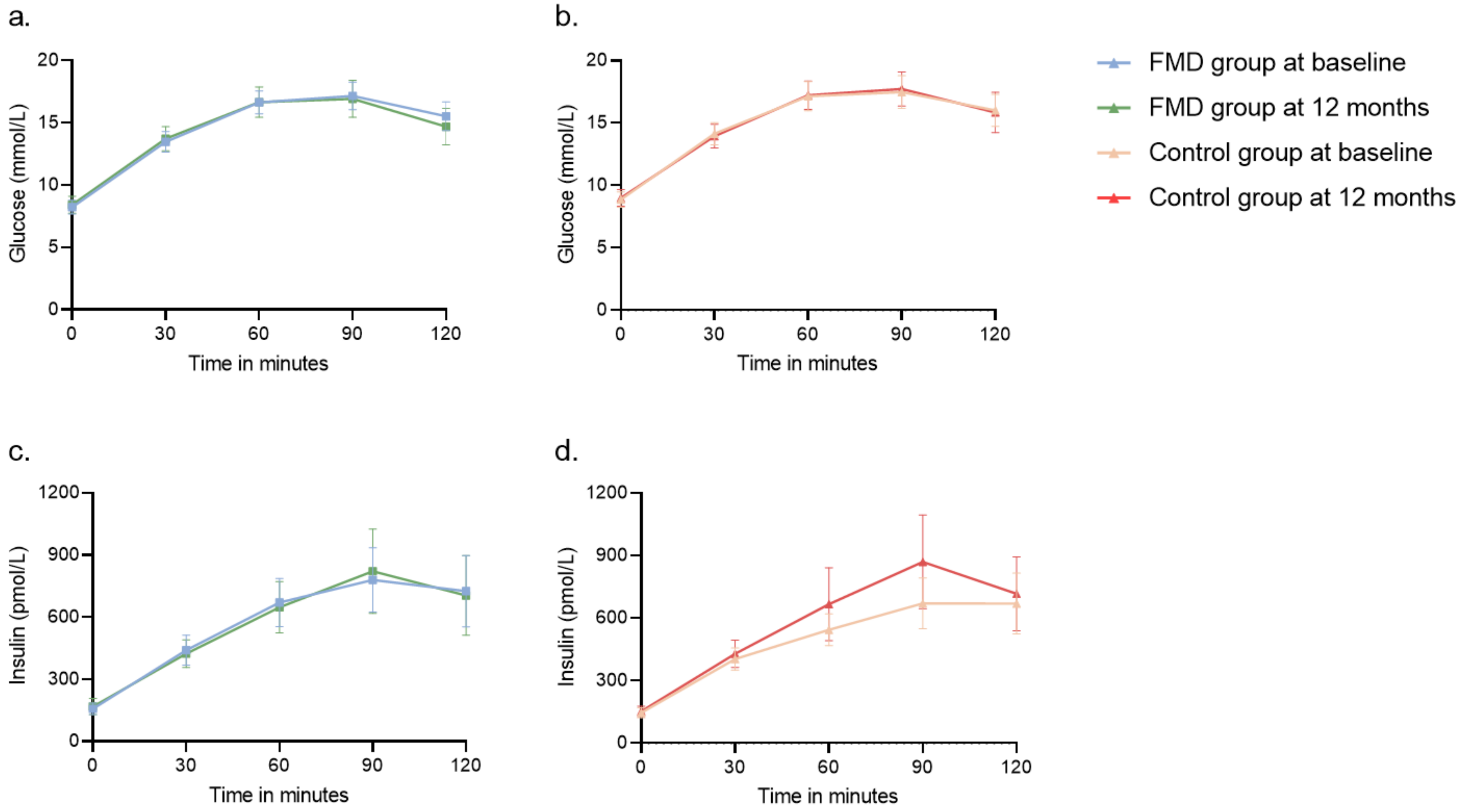

Lines represent the mean levels of glucose or insulin; error bars represent the 95% confidence intervals.
a) Glucose measurements of the FMD group (*n*=44 at baseline; *n*=39 at 12 months). b) Glucose measurements of the control group (*n*=39 at baseline; *n*=32 at 12 months).
c) Insulin measurements of the FMD group (*n*=44 at baseline; *n*=39 at 12 months). d) Insulin measurements of the control group (*n*=39 at baseline; *n*=32 at 12 months).
FMD = fasting-mimicking diet.

ESM Figure 3. Glucose-lowering medication use, HbA_{1c}, and glycaemic management at 12 months (per protocol analyses)

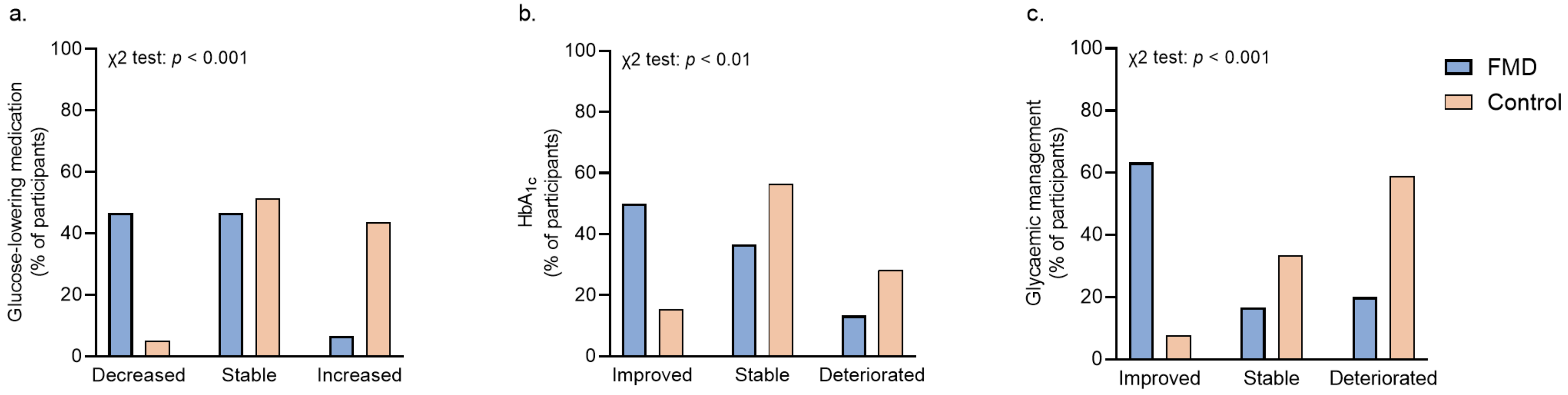

Plotted bars represent percentage of participants. Differences between FMD group and control group were evaluated using the Chi square test. Number of participants with data available at baseline and 12 months were used for each outcome.

A) Change in glucose-lowering medication, with decrease defined as a lower dose, stable as no change and increased as higher dose or other class of glucose-lowering medication at the end of the study compared to baseline. FMD *n*=30, controls *n*=39.

(B) Change in HbA_{1c}, defined as follows; Improved: an HbA_{1c} that is ≥0.5 % (≥5 mmol/mol) lower at the end of the study compared to baseline. Stable: a change in HbA_{1c} of <0.5 % (<5 mmol/mol) at the end of the study compared to baseline. Deteriorated: an HbA_{1c} that is ≥0.5 % (≥5 mmol/mol) higher at the end of the study compared to baseline. FMD *n*=30, controls *n*=39.

(C) Glycaemic management, defined as follows; Improved: a lower dose or class of glucose-lowering medication with an HbA_{1c} not more than 0.5 % (5 mmol/mol) higher at the end of the study compared to baseline or; no change in glucose-lowering medication with an HbA_{1c} that is ≥0.5 % (≥5 mmol/mol) lower at the end of the study compared to baseline. Stable: no change in glucose-lowering medication use and a difference in HbA_{1c} of <0.5 % (<5 mmol/mol) at the end of the study compared to baseline. Deteriorated: a higher dose or class of glucose-lowering medication at the end of the study compared to baseline or; an HbA_{1c} that is ≥0.5 % (≥5 mmol/mol) higher at the end of the study compared to baseline with no change in glucose-lowering medication (table 1). FMD *n*=30, controls *n*=39.

FMD=fasting-mimicking diet. HbA_{1c}=glycated haemoglobin.
